# Epigallocatechin-3-Gallate Suppresses Human Herpesvirus 8 Replication and Induces ROS Leading to Apoptosis and Autophagy in Primary Effusion Lymphoma Cells

**DOI:** 10.3390/ijms19010016

**Published:** 2017-12-21

**Authors:** Ching-Yi Tsai, Chang-Yu Chen, Yee-Hsuan Chiou, Huey-Wen Shyu, Kuan-Hua Lin, Miao-Chen Chou, Mei-Han Huang, Yi-Fen Wang

**Affiliations:** 1 Department of Medical Laboratory Science and Biotechnology, Fooyin-University, Kaohsiung 83102, Taiwan; a07646815@yahoo.com.tw (C.-Y.T.); chencyallen1689@gmail.com (C.-Y.C.); shyuhw@hotmail.com (H.-W.S.); su6473@mail2000.com.tw (K.-H.L.); miaochen119@gmail.com (M.-C.C.); hmhmhtw8@gmail.com (M.-H.H.); 2 Department of Pediatrics, Kaohsiung Veterans General Hospital, Kaohsiung 83102, Taiwan; chysn@ms6.hinet.net

**Keywords:** EGCG, primary effusion lymphoma, ROS, apoptosis, autophagy

## Abstract

Epigallocatechin-3-gallate (EGCG), the major constituent of green tea, has been shown to induce cell death in cancer cells. Primary effusion lymphoma (PEL) is an aggressive neoplasm caused by human herpesvirus 8 (HHV8). In this study, we examined the role of EGCG on PEL cells in cell death and HHV8 replication. We performed trypan blue exclusion assay to assess the cell viability of PEL cells, flow cytometry analysis to examine the cell cycle distribution and reactive oxygen species (ROS) generation, caspase-3 activity to assay apoptosis, acridine orange staining to determine autophagy, and immunoblotting to detect the protein levels involved in apoptosis and autophagy as well as mitogen activated protein kinases (MAPKs) activation upon EGCG treatment. The expression of the HHV8 lytic gene was determined by luciferase reporter assay and reverse transcription-PCR, and viral progeny production was determined by PCR. Results revealed that EGCG induced cell death and ROS generation in PEL cells in a dose-dependent manner. *N*-acetylcysteine (NAC) inhibited the EGCG-induced ROS and rescued the cell from EGCG-induced cell death. Even though EGCG induced ROS generation in PEL cells, it reduced the production of progeny virus from PEL cells without causing HHV8 reactivation. These results suggest that EGCG may represent a novel strategy for the treatment of HHV8 infection and HHV8-associated lymphomas.

## 1. Introduction

Primary effusion lymphoma (PEL) is caused by the clonal expansion of human herpesvirus 8 (HHV8; also known as Kaposi’s sarcoma-associated herpesvirus)-infected B cells [1]. HHV8 is an oncogenic herpesvirus that pathogenically infects endothelial cells and B lymphocytes. Moreover, HHV8 is also implicated in the pathogenesis of Kaposi’s sarcoma and multicentric Castleman’s disease [2,3].

The replicative cycle of HHV8 exists as latency and lytic replication. During latency, only a restricted subset of viral transcripts is expressed (including LANA, v-FLIP, and v-cyclin) that contributes to HHV8-associated malignancies by manipulating cell proliferation and apoptosis [2,4]. Lytic replication is required for HHV8 production and propagation. Similar to other herpesviruses, latent HHV8 can be reactivated to undergo lytic replication. The HHV8 replication and transcription activator (RTA) is the key viral regulator of HHV8 reactivation [5,6]. PEL cells are predominantly infected with a latent form of HHV8 [4]. Extensive studies have shown that HHV8 targets multiple pathways to induce cell proliferation and survival for promoting tumor development [2].

PEL is a rare and aggressive non-Hodgkin lymphoma with a short (usually less than 6 months) median survival time upon diagnosis due to resistance to conventional chemotherapy [7]. Lack of sufficient potency and specificity of the current chemotherapy-based regimens for PEL treatment leads to a pressing need for specific drug development.

Epigallocatechin-3-gallate (EGCG) is the most abundant and active compound found in green tea and has gained much attention for its anticancer and antivirus effects [8]. There are ample evidences that show the potential chemotherapeutic efficacy of EGCG against cancers of the skin, lung, breast, colon, liver, stomach, and prostate [9]. EGCG is auto-oxidized in both cells and culture media, and oxidized EGCG induces apoptosis in cells. Recent studies demonstrate that EGCG can induce preferential death in cancer cells due to the cancer-specific induction of ROS (reactive oxygen species) [10]. EGCG was also shown to block Epstein-Barr virus (EBV)-induced transformation and the expression of viral lytic proteins [11,12]. However, the effects of EGCG on PEL cells and HHV8 replication in PEL cells have not yet been reported.

In this study, we investigated the antitumor and antiviral activity of EGCG against human primary effusion lymphoma cells. Our data demonstrate that EGCG induces cell death in PEL cells via a mechanism involving the generation of ROS. We also examined the effects of EGCG on HHV8 replication and reactivation. Even though EGCG induced ROS generation in PEL cells, it reduced the production of progeny virus from PEL cells without causing HHV8 reactivation. These results suggest that EGCG may represent a novel strategy for the treatment of HHV8 infection and HHV8-associated lymphomas.

## 2. Results

### 2.1. EGCG Inhibited the Growth of HHV8-Harboring PEL Cells

**Figure 1:**
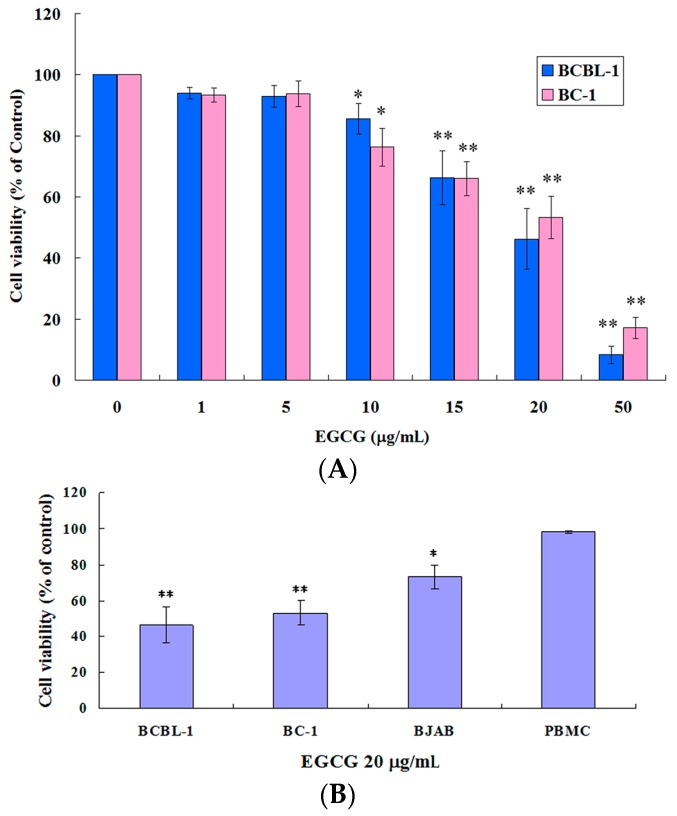
Epigallocatechin-3-gallate (EGCG) reduced cell viability in human herpesvirus 8 (HHV8)-harboring primary effusion lymphoma (PEL) cells. (**A**) PEL cells (BCBL-1 and BC-1) were treated with EGCG at the concentrations indicated for 24 h; (**B**) Sensitivity of BCBL-1, BC-1, BJAB cells and PBMCs to EGCG. BCBL-1, BC-1, BJAB cells and PBMCs were treated with 20 μg/mL of EGCG for 24 h. Cell viability was determined by the trypan blue exclusion assay. The cells in supplemented medium were used as a control. The values represent mean ± SE of three independent experiments and are presented as the percentage of the control. * *p* < 0.05 and ** *p* < 0.01 indicate significant differences between the control and EGCG-treated cells.

To determine the effects of EGCG on the PEL cells, two PEL cell lines including BCBL-1 (HHV8-positive) and BC-1 (HHV8-positive and EBV-positive) were treated with 0, 1, 5, 10, 15, 20, 50 μg/mL EGCG for 24 h. The cellular viability was assessed using trypan blue exclusion assay. Both HHV8 containing PEL cell lines were susceptible to EGCG in a dose-dependent manner (Figure 1A). Results of the trypan blue exclusion assays directly paralleled those obtained by alamarBlue assay. The effect of EGCG on a HHV8-negative and EBV-negative lymphoma cell line (BJAB) and peripheral blood mononuclear cells (PBMCs; isolated from laboratory volunteers) was also detected. EGCG showed less effect on cell viability of the HHV8-negative EBV-negative lymphoma cells and had no inhibitory effect on the PBMCs (Figure 1B).

### 2.2. EGCG Induced G_2_-M Arrest and Apoptosis in PEL Cells

**Figure 2:**
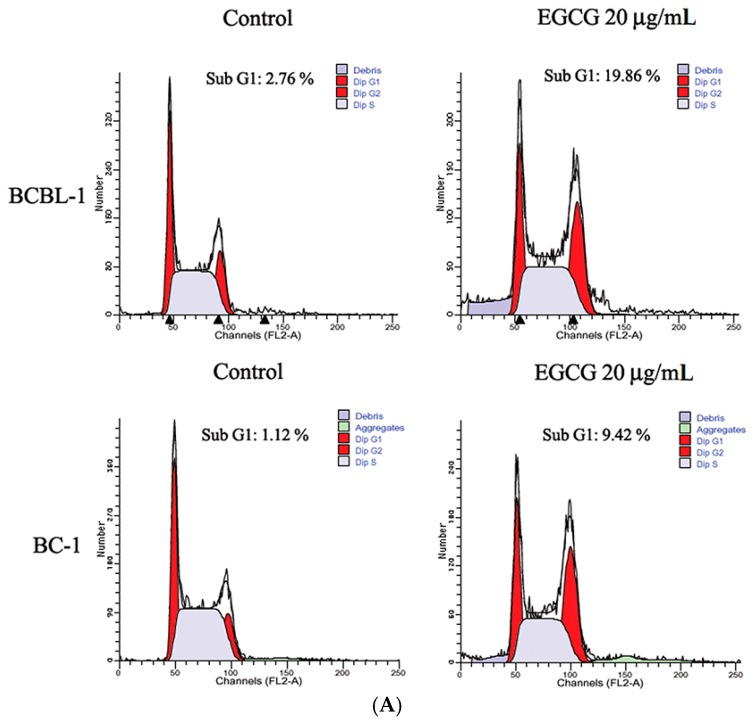

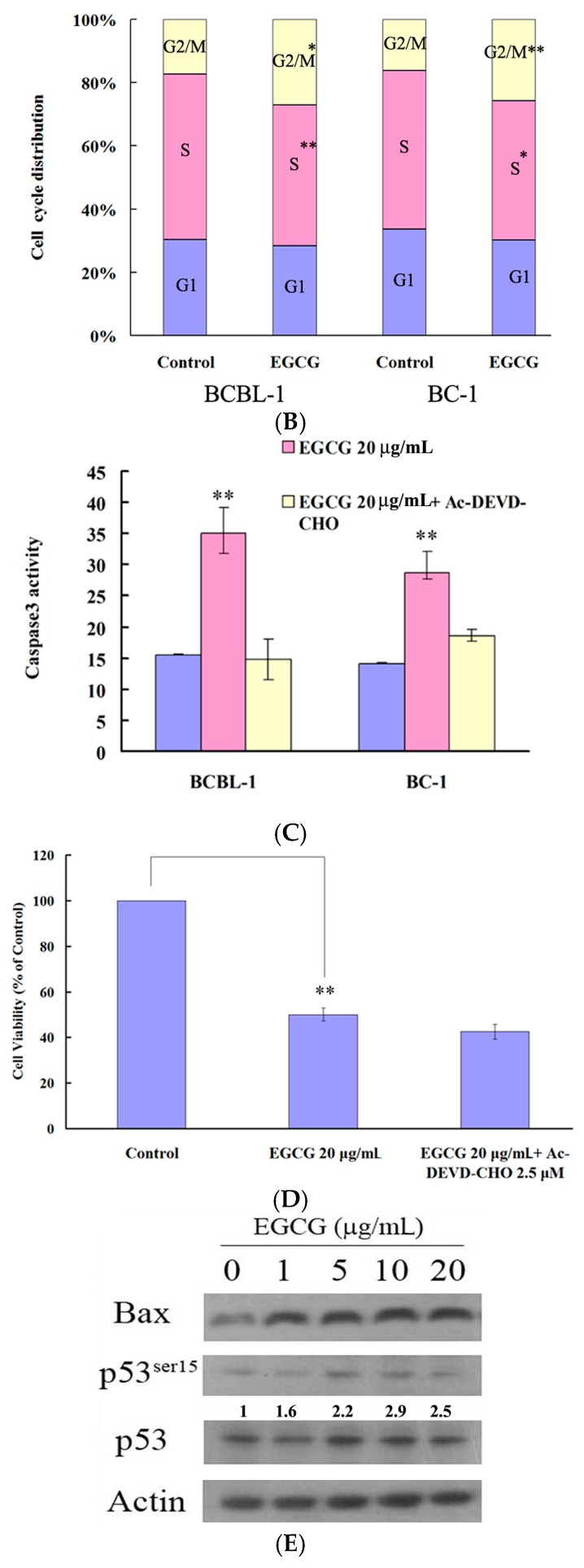
EGCG induces cell cycle arrest and apoptosis in PEL cells. (**A**) BCBL-1 and BC-1 cells were untreated or treated with 20 μg/mL EGCG for 24 h. After treatment, PEL cells were incubated in methanol, treated with propidium iodide and subjected to cell cycle analysis using a Becton Dickinson FACScan flow cytometer and ModFit software described in the Materials and Methods section. Results are shown as the percentage of the apoptotic cells (sub-G1) in the EGCG-treated PEL cells; (**B**) Cell cycle distribution of EGCG-treated PEL cells. Representative results of the actual cell cycle profile are shown; (**C**) EGCG induced caspase-3 activation in PEL cells; (**D**) Effects of caspase-3 inhibitor (Ac-DEVD-CHO) on the cell viability of EGCG-treated BCBL-1 cells. The values represent mean ± SE of three independent experiments and are presented as the percentage of the control; * *p* < 0.05 and ** *p* < 0.01 indicate significant differences between the control and EGCG-treated cells. (**E**) Western blot analysis to detect p53 activation and Bax expression in EGCG-treated BCBL-1 cells. The representative data are shown. The relative intensity of phosphor-p53 at Ser15/total p53 is shown under each blot.

To elucidate whether EGCG-induced cell growth inhibition is mediated via alterations in cell cycle progression, we evaluated its effect on cell cycle phase distribution by flow cytometric studies. As shown in Figure 2A,B, DNA flow cytometric analysis indicated that EGCG caused a significant G_2_-M arrest in PEL cells. Moreover, the percentage of hypodiploid cells (i.e., sub-G_1_ fraction) increased in EGCG-treated PEL cells compared with control cells (Figure 2A). To examine the contribution of an apoptotic event in EGCG-induced decline of PEL cells viability, caspase-3 activation was determined. Results revealed that EGCG induced caspase-3 activation in PEL cells, and caspase inhibitor could attenuate EGCG-induced caspase-3 activity (Figure 2C). However, caspase inhibitor failed to rescue the cells from EGCG-induced PEL cell death (Figure 2D). These results indicate that EGCG induces cell cycle arrest in the G_2_-M phase and apoptosis in PEL cells, but EGCG inhibition of PEL cell growth may not be restricted to apoptosis.

Previous studies have demonstrated that chemical activation of p53 in PEL cells is sufficient to induce the expression of p53 target genes and lead to cell growth inhibition and apoptosis [13]. To evaluate whether EGCG could induce p53 activation, the p53 phosphorylation on serine 15 and p53 downstream gene Bax was detected by Western blot analysis. Results showed that the EGCG treatment caused p53 activation and increased the expression of Bax (Figure 2E).

### 2.3. EGCG Induced Autophagy in PEL Cells

**Figure 3:**
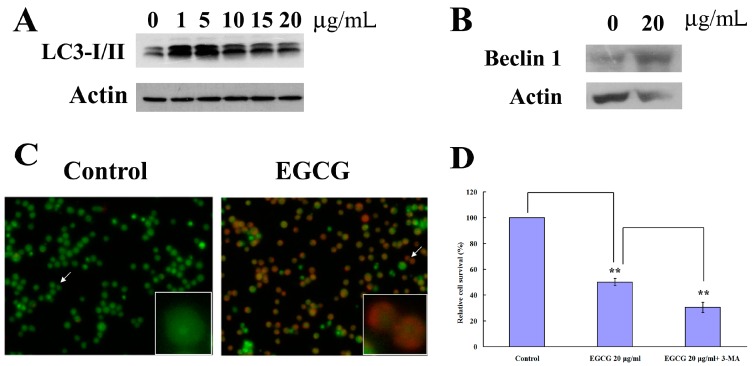
EGCG induced autophagy in PEL cells. (**A**) Western blot analysis to detect LC3-I/II expression in EGCG-treated BCBL-1 cells; (**B**) Western blot analysis to detect Beclin expression in EGCG-treated BCBL-1 cells; (**C**) EGCG induced formation of acidic vesicular organelles in PEL cells; (**D**) Effects of autophagy inhibitor (3-MA) on the cell viability of EGCG-treated BCBL-1 cells. The representative data are shown. The results are the means of three independent experiments, and bars represent the standard errors. ** *p* < 0.01 indicates significant differences between the control and EGCG-treated cells, or significant differences between the cells treated with EGCG and the cells cotreated with EGCG and 3-MA.

Previous studies have shown that EGCG induced autophagy, and the suppression of autophagy enhanced EGCG-induced cell death in human mesothelioma cells [14]. Therefore, we examined whether EGCG could induce autophagy in PEL cells. Microtubule-associated protein light chain 3 (LC3) is well known to monitor autophagy [15]. Results showed that EGCG caused LC3 transition in a concentration-dependent manner in PEL cells (Figure 3A). To confirm the induction of autophagy, we measured the expression of Beclin-1. Results revealed that EGCG could induce the expression of Beclin-1 (Figure 3B). Acridine orange (AO) is a marker of acidic vesicular organelle (AVOs) that fluoresces green in the whole cell except in acidic compartments (mainly late autophagosomes), where it fluoresces red. Development of AVOs is a typical feature of autophagy, and its formation indicates the maturation of autophagosomes and an efficient autophagic process, since only mature/late autophagosomes are acidic. By AO staining, red fluorescent spots appeared on EGCG-treated PEL cells, while the control cells showed mainly green cytoplasmic fluorescence (Figure 3C). We further examined whether the inhibition of autophagy affected the EGCG-induced cell death in PEL cells. PEL cells were pretreated with autophagy inhibitor 3-Methyladenine (3-MA) (3 mM) for 1 h, and then cotreated with EGCG (20 μg/mL) for 24 h. Next, the cell viability was analyzed by trypan blue exclusion assay. 3-MA failed to rescue cell death in EGCG-treated PEL cells (Figure 3D). Similar to the previous observation in human mesothelioma cells [14], our data also indicate that the inhibition of autophagy enhances EGCG-induced cell death in PEL cells.

### 2.4. EGCG Induced Apoptosis and Autophagy through ROS Generation

**Figure 4:**
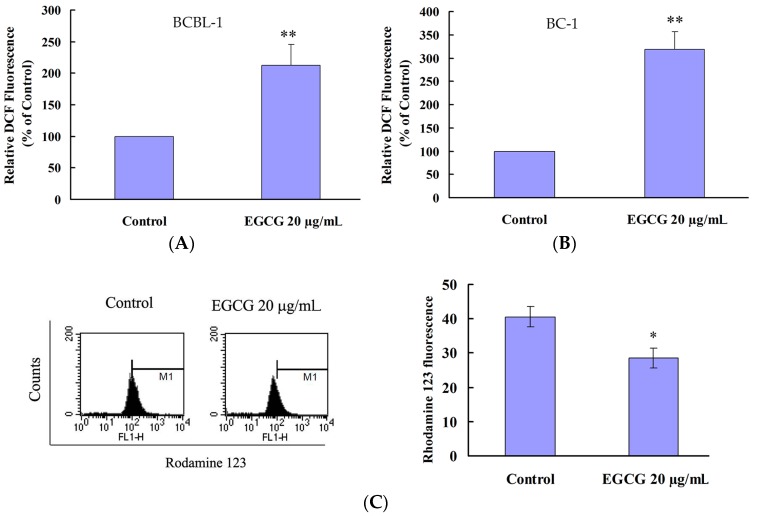
EGCG induced oxidative stress in HHV8-harboring PEL cells. (**A**) BCBL-1 and (**B**) BC-1 were treated with 20 μg/mL EGCG for 24 h. ROS generation was detected by staining with H_2_DCFDA; (**C**) The mitochondrial membrane potential (MMP) was monitored by staining with Rh123 dye. Results are expressed as mean Rh123 fluorescence. The representative data are shown. The values represent mean ± SE of three independent experiments and are presented as the percentage of the control. * *p* < 0.05 and ** *p* < 0.01 indicate significant differences between the control and EGCG-treated cells.

Previous studies have demonstrated that the ROS level is increased in EGCG-treated cancer cells but not in normal cells [10]. In order to determine whether EGCG treatment could induce oxidative stress in PEL cells, the intracellular ROS level was measured using a fluorescence probe (H_2_DCFDA). The results showed that 20 μg/mL EGCG could enhance the ROS level in both BCBL-1 (Figure 4A) and BC-1 cells (Figure 4B). Apoptosis is closely related to the decline of mitochondrial membrane potential (MMP) since mitochondria play fundamental role in oxidative defense. Thus, the loss of MMP in EGCG-treated BCBL-1 cells was determined using Rhodamine 123 dye at 24 h. As expected, the loss of MMP was observed in EGCG-treated PEL cells (Figure 4C).

**Figure 5:**
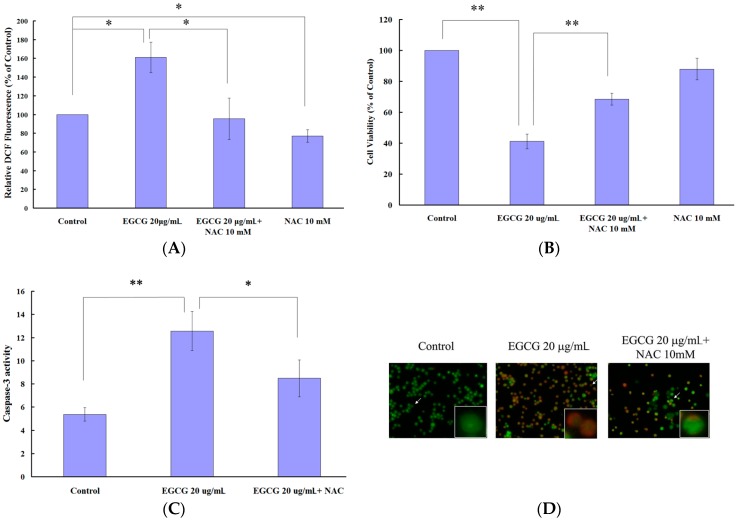
*N*-acetylcysteine attenuated the EGCG-induced ROS generation, cytotoxicity, caspase-3 activation, and formation of acidic vesicular organelles in PEL cells. PEL cells were preincubated with or without *N*-acetylcysteine (NAC) for 1 h before treatment with 20 μg/mL EGCG for 24 h. (**A**) The ROS generation was detected by staining with H_2_DCFDA; (**B**) The cell viability was determined by trypan blue exclusion assay; (**C**) The caspase-3 activity assay was evaluated by using the CaspACE Assay System, Colorimetric (Promega); (**D**) The formation of acidic vesicular organelles (AVOs) was observed by acridine orange staining. The results are the means of three independent experiments, and bars represent the standard errors. The representative data are shown. * *p* < 0.05 and ** *p* < 0.01 indicates significant differences between the control and EGCG-treated cells.

To elucidate whether ROS generation is involved in EGCG-induced cell death, EGCG-treated PEL cells were cotreated with *N*-acetylcysteine (NAC), a known ROS scavenger, for 24 h. NAC reduces EGCG-induced ROS generation in PEL cells when compared with cells treated with EGCG alone (Figure 5A). NAC also attenuated EGCG-induced cytotoxicity in PEL cells (Figure 5B). In addition, cotreatment of NAC inhibited the EGCG-induced caspase-3 activation (Figure 5C) and autophagy (Figure 5D) in PEL cells.

### 2.5. EGCG Induced Mitogen-Activated Protein Kinase Activation in PEL Cells

**Figure 6:**
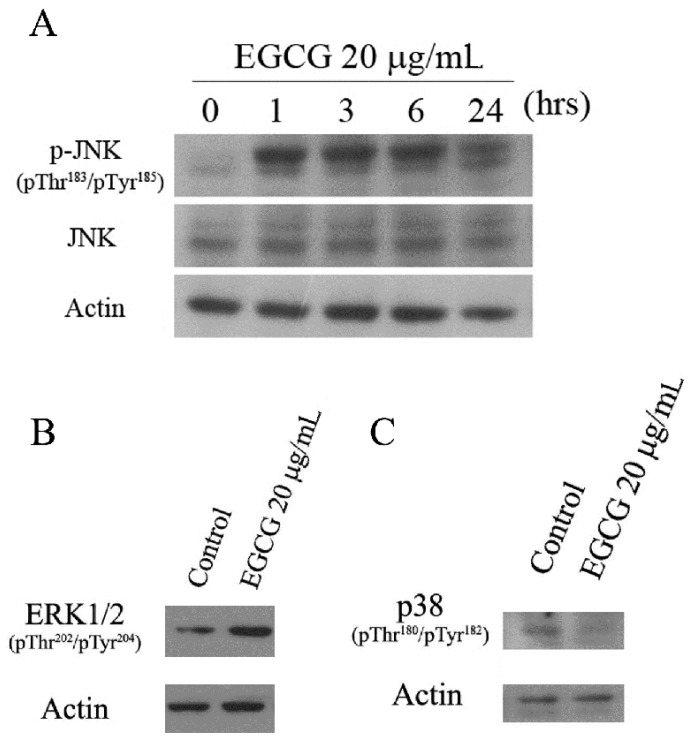
(**A**) Western blot analysis of JNK activation in EGCG-treated BCBL-1 cells. BCBL-1 cells were treated with 20 μg/mL of EGCG for 1, 3, 6, and 24 h. The protein levels of JNK and phosphorylated forms of JNK were determined; (**B**) Western blot analysis of ERK activation in EGCG-treated BCBL-1 cells; (**C**) Western blot analysis of p38 inactivation in EGCG-treated BCBL-1 cells. BCBL-1 cells were treated with 20 μg/mL of EGCG for 24 h. The protein levels of phosphorylated forms of ERK and p38 were determined. β-actin was used as the loading control.

Mitogen-activated protein kinases (MAPKs), including c-Jun NH2-terminal kinases (JNK1⁄2), extracellular signal-regulated kinase (ERK1/2), and p38 MAP kinase, are involved in the response to the intracellular redox state and oxidative stress, and potentially affect cell survival or cell death [16,17]. Previous studies have reported that EGCG induced the activation of JNK and p38 in human leukemic cells [18]. To evaluate the effects of EGCG on MAP kinase signaling in PEL cells, we determined the activation of MAP kinases by Western blot analysis. As shown, the phosphorylation of JNK1/2 was markedly induced by EGCG in PEL cells (Figure 6A). ERK1/2 was also activated by EGCG treatment (Figure 6B), but the phosphorylation of p38 was reduced upon EGCG treatment in PEL cells (Figure 6C).

### 2.6. EGCG Inhibited HHV8 Replication in PEL Cells

**Figure 7:**
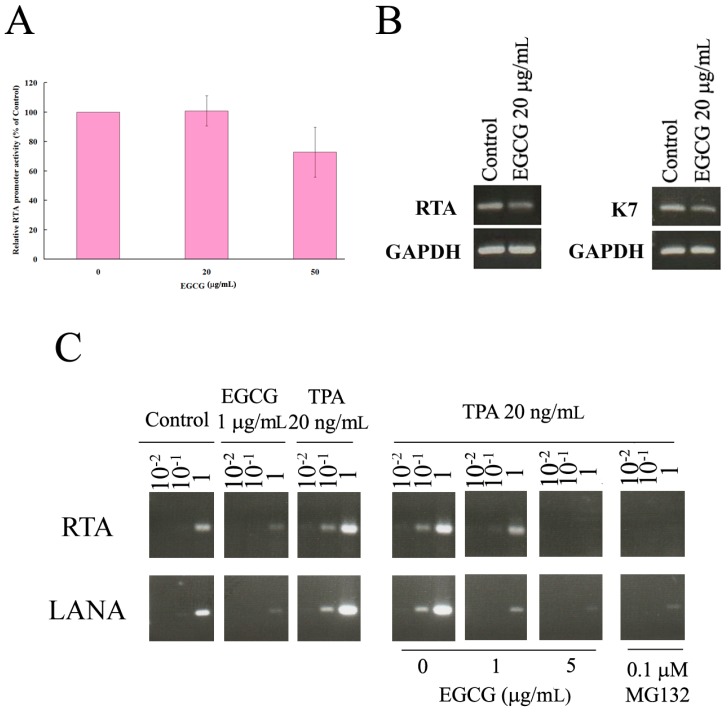
EGCG treatment inhibited HHV8 virus replication in PEL cells. (**A**) EGCG treatment failed to induce RTA promoter activity. 293T cells were co-transfected with 50 ng of DNA encoding RTA promoter and 2 ng of pTK-RL plasmid DNA (encoding *Renilla* luciferase). The RTA promoter activities were determined by the dual-luciferase reporter assay kit (Promega, Madison, WI, USA) in untreated 293T cells or 293T cells treated with EGCG (20 μg/mL or 50 μg/mL) for 24 h; (**B**) HHV8 lytic genes (RTA and K7) expression levels in EGCG-treated PEL cells were detected by RT-PCR; (**C**) BCBL-1 cells were treated with 1 μg/mL EGCG or 20 ng/mL TPA for 48 h, or incubated with TPA for 24 h to induce the production of new virus particles, and then incubated with or without EGCG (1 μg/mL and 5 μg/mL) or MG132 (0.1 μg/mL) for 24 h. The serial dilutions (10^−2^, 10^−1^ and 1×) of viral genomes extracted from culture medium were quantified by PCR using HHV8 genes (RTA and LANA). The representative data are shown.

It has been reported that valproic acid induces the apoptosis of PEL cells, accompanied by HHV8 reactivation [19]. To examine whether EGCG induces HHV8 reactivation in PEL cells, the RTA promoter reporter plasmid was utilized to analyze the effect of EGCG on the HHV8 lytic gene expression in BCBL-1 cells. Results showed that 20 μg/mL EGCG had no effect on RTA promoter activity but 50 μg/mL EGCG reduced RTA promoter activity (Figure 7A). However, the decrease of RTA promoter activity between untreated and 50 μg/mL EGCG treatment showed no statistically significant difference (*p* = 0.0751). Reverse transcription-PCR results confirmed that 20 μg/mL EGCG did not induce the gene expression of RTA but slightly reduced the gene expression of K7, a HHV8 anti-apoptosis gene expressed at the lytic cycle (Figure 7B).

HHV8 is crucial for survival of PEL cells [20]. To evaluate whether EGCG interferes with HHV8 replication, BCBL-1 cells were cultured in EGCG alone or in tetradecanoylphorbol acetate (TPA)-containing media to induce lytic viral replication in the presence or absence of EGCG for 48 h. Culture medium containing capsidated viral particles was analyzed by PCR to quantify viral DNA. Previous studies have demonstrated that proteasome inhibitors, including MG132, decreased the production of progeny virus from PEL cells [21]. MG132 was used as a control to test the effects of EGCG on HHV8 viral progeny production. Results showed that low concentration of EGCG (1 μg/mL) alone suppressed viral particle production (Figure 7C). Similar to MG132, EGCG also inhibited the TPA-induced HHV8 reactivation (Figure 7C).

## 3. Discussion

**Figure 8:**
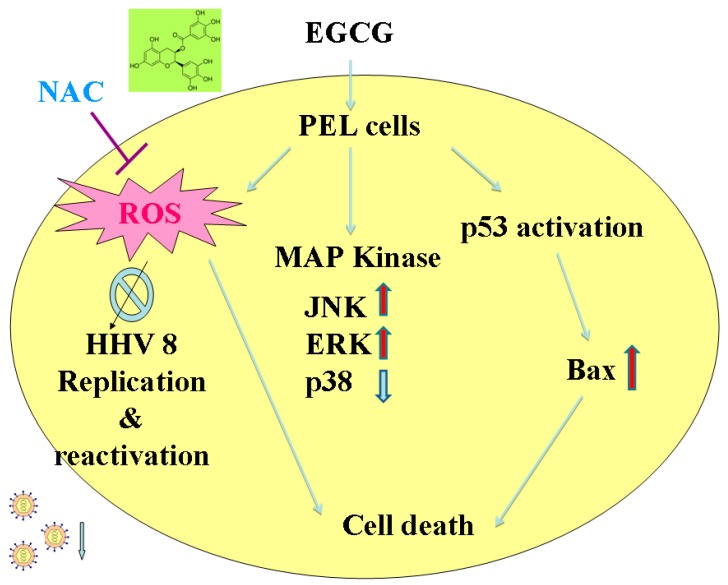
Schematic summary of EGCG-induced cell death in HHV8-harboring PEL cells.

Green tea is the most widely consumed beverage in the world. Previous studies have reported that consumption of green tea has benefits for the prevention and treatment of various diseases, including cancers [22]. EGCG is the most abundant tea catechin in green tea and possesses a variety of pharmacological properties, such as chemopreventive, anti-carcinogenic, anti-infective, and antioxidant activities [8,22]. There are many reports about the effects of EGCG on cancer cell growth. However, the effects of EGCG on HHV8-harboring PEL cells have not been investigated. In this study, we demonstrate that EGCG inhibits the proliferation of HHV8-harboring PEL cells but proves to be non-toxic to PBMCs derived from healthy volunteers. EGCG can increase oxidative stress in HHV8-harboring PEL cells and cotreatment with NAC attenuates the EGCG-induced generation of ROS and cell death, indicating that ROS is partially involved in EGCG-induced PEL cell death. In addition, EGCG can inhibit the replication and reactivation of HHV8 in PEL cells. Since HHV8 is essential for PEL survival [20], we propose that EGCG leads to PEL cell death by inhibiting HHV8 replication and inducing ROS generation. The possible mechanism is summarized in Figure 8. These data indicate that EGCG may be a potential treatment for aggressive PEL and HHV8 infection.

EGCG contains antioxidant as well as pro-oxidant properties. Evidences in the literature suggest that the prooxidant activity of EGCG may account for its anti-proliferative and cancer therapeutic effects [23]. We observe that EGCG induces ROS generation and the loss of mitochondrial membrane potential (Figure 4) in PEL cells, leading to apoptosis and autophagy. Cotreatment of NAC decreases the EGCG-induced ROS production and also attenuates EGCG-induced apoptosis and autophagy (Figure 5), suggesting that the prooxidant activity of EGCG is partially related to the anticancer effects of EGCG in HHV8-harboring PEL cells. In addition, EGCG shows selective cytotoxicity in PEL cells and is not toxic to PBMCs (Figure 1B). Recent studies have reported that EGCG can behave as a prooxidant in the presence of Cu(II), leading to cytotoxicity [24]. Since copper levels are increased in various malignancies, the copper-dependent prooxidant cytotoxic mechanism may explain the anticancer effects and preferential cytotoxicity of EGCG.

Previous studies reported that the inhibitory concentration (IC50) of EGCG at 36 h for lymphoma cells Jeko-1 and Raji was 57.98 and 61.24 μg/mL, respectively [25]. We have shown that EGCG has anti-proliferative effects against PEL cells at a much lower concentration. After treatment with 20 μg/mL EGCG for 24 h, the cell viability of both PEL cells (BCBL-1: 39.19%, BC-1: 47.72%) is much lower compared with that of BJAB cells (72.51%) (Figure 1B). These data suggest that PEL cells are more susceptible to EGCG treatment compared with other lymphoma cells.

It has been reported that EGCG induces both apoptosis and autophagy in oral cancer cells, suggesting that EGCG can target multiple cell death pathways [26]. We observed that EGCG induced caspase-3 activation and increased the Bax expression in PEL cells (Figure 2C,E), indicating that EGCG can induce apoptosis in HHV8-harboring PEL cells. Meanwhile, EGCG treatment also increased the formation of acidic vesicular organelles and the expression of LC3-I/II and beclin-1 (Figure 3), revealing that EGCG can also induce autophagy in HHV8-harboring PEL cells. However, caspase-3 inhibitor (Figure 2D) and autophagy inhibitors (3-MA) (Figure 3C) failed to rescue the cytotoxic effect of EGCG on PEL cells. Autophagy plays dual roles in cancer; it can support the survival of cancer cells as well as promote cell death [27]. The autophagy inhibitors (3-MA) (Figure 3C) slightly increased the cytotoxicity of EGCG and the cotreatment of 3-MA and caspase-3 inhibitor augmented EGCG-induced cell death [28] that autophagy protects PEL cells from death. Cotreatment of NAC attenuated the EGCG-induced caspase-3 activation and AVOs formation in PEL cells (Figure 5), suggesting that EGCG-induced apoptosis and autophagy are partially due to the production of ROS.

Necroptosis is a form of nonapoptotic cell death driven by the receptor interacting protein kinase1 and 3 (RIP1 and RIP3) and can be specifically inhibited by necrostatin-1 [29]. We have also examined the effects of necrostatin-1 on cell death in EGCG-treated PEL cells. Results showed that necrostatin-1 did not attenuate but increased EGCG-induced cell death in PEL cells (supplementary data Figure S1). Kreuzaler and Watson reported that autophagy, lysosomal-mediated programmed cell death, and necroptosis can serve both as a backup to apoptosis as well as preferred death pathways in certain cell types [29]. Our results suggest that multiple and more complicated cell death pathways are involved in EGCG-induced cell death in PEL cells.

Previous studies have demonstrated that JNK, ERK, and p38 (MAP kinase pathways) are important for theHHV8 productive replication cycle in both primary infection and reactivation from latency [30]. Inhibitors of MAP kinase pathways decrease the production of virion progeny from TPA-induced HHV8 reactivation [30]. In Figure 6, results show that EGCG treatment can induce ERK and JNK activation, but inhibit the activation of p38. It has been reported that ROS are required for HHV8 reactivation and p38 signaling is essential for HHV8 reactivation induced by ROS [31]. Our results display that EGCG treatment induces ROS generation in PEL cells (Figure 4). However, EGCG treatment fails to lead to HHV8 reactivation, but inhibits HHV8 virus progeny production (Figure 7). Moreover, EGCG blocks TPA-induced HHV8 reactivation (Figure 7). It has been reported that celecoxib inhibits the lytic activation of HHV8 via the downregulation of RTA expression, by inhibiting the activation of p38 MAPK [32]. Our results indicate that the decrease of p38 activation may inhibit the HHV8 reactivation in EGCG-treated PEL cells. It also suggests that EGCG-induced cell deat0h in HHV8-harboring PEL cells is mediated by multiple pathways and the mechanisms of EGCG-induced PEL cell death are complicated. Whether EGCG can affect the expression profiles of HHV8 microRNAs is currently been investigated.

Activation of the JNK pathway is implicated in EGCG-induced apoptosis in various cancer cells [23]. In Figure 7, results show that EGCG treatment induces JNK activation in a time-dependent manner. However, cotreatment of the JNK inhibitor, SP600125, fails to attenuate EGCG-induced cell death, but rather increases the cytotoxic effect of EGCG (supplementary data Figure S2). The roles of JNK activation in EGCG-induced PEL cell death remain to be clarified.

It has been reported that EGCG possesses antiviral effects against different viruses, including EBV and Human immunodeficiency virus (HIV) [33]. EGCG can reduce the expression of EBV lytic genes and inhibits the production of HIV p24 antigen [33,34,35,36]. HHV8 is essential for PEL cell survival [20]. Our results showed that EGCG inhibited the RTA expression and reduced HHV8 virus progeny production (Figure 7), indicating that EGCG has anti-HHV8 activity.

In summary, EGCG suppresses HHV8 replication and induces ROS, leading to apoptosis and autophagy in PEL cells. These finding suggests that EGCG can be a potential anti-HHV8 drug and may be considered as an effective treatment for HHV8-related tumors.

## 4. Materials and Methods

### 4.1. Cell Culture

BCBL-1 (ATCC CRL11982; HHV8-positive and EBV-negative) and BC-1 (ATCC CRL-2230; HHV8-positive and EBV-positive) cells are primary effusion lymphoma (PEL) cells. BJAB (ATCC HB-136) cells are HHV8-negative and EBV-negative human lymphoblastoid cells. All of the lymphoma cell lines were grown in RPMI 1640 (Invitrogen, Carlsbad, CA, USA), supplemented with 10% heat-inactivated fetal bovine serum (HyClone, Logan, UT, USA), 2 mM l-glutamine, 100 units/mL penicillin, and 100 units/mL streptomycin (Invitrogen). Cells were grown at 37 °C in a humidified 5% CO_2_ atmosphere.

### 4.2. Reagents

EGCG, *N*-acetylcysteine (NAC), *N*-acetyl-Asp-Glu-Val-Asp-al (Ac-DEVD-CHO), and 3-Methyladenine (3-MA) were purchased from Sigma-Aldrich Chemical Co. (St. Louis, MO, USA). EGCG was dissolved in H_2_O at 5 mg/mL as a stock solution. NAC was dissolved in phosphate buffered saline (PBS) buffer at 1 M as a stock solution. The caspase 3 inhibitor (Ac-DEVD-CHO) was dissolved in H_2_O at 1 mg/mL as a stock solution. 3-MA was dissolved in H_2_O at 30 mg/mL as a stock solution. Cells were pretreated with NAC, Ac-DEVD-CHO, 3-MA, or necrostatin-1 for 1 h prior to EGCG treatment.

### 4.3. Cell Viability Assays

The effect of EGCG on PEL cell viability was determined by trypan blue exclusion assay. Cells suspended at 2 × 10^5^ cells/mL were incubated with various concentrations of EGCG and plated at a density of 2 × 10^5^ cells per well in 24-well plates for 24 h. Cell viability was determined by trypan blue exclusion assay. The untreated cells were utilized as the control, and the cell viability was compared with the control. Each treatment was performed in triplicate and three independent experiments were performed. Error bars represent the standard errors.

### 4.4. Cell Cycle and Sub-G1 Analysis

Cell cycle distributions and sub-G1 cells were determined by propidium iodide (PI, Sigma-Aldrich Chemical Co., Ex/Em = 488 nm/617 nm) staining. PEL cells were seeded at a concentration of 2 × 10^5^ cells/mL and incubated with EGCG (20 μg/mL) for 24 h. Cells were harvested by centrifugation, fixed with methanol at −20 °C for 18 h, and finally treated with RNase (100 μg/mL) and propidium iodine (50 μg/mL) at room temperature for 30 min. Cell cycle distributions and sub-G1 cells were measured with a FACSCalibur (Becton Dickinson, Franklin Lakes, NJ, USA) and analyzed using Cell Quest (Becton Dickinson, Franklin Lakes, NJ, USA). 

### 4.5. Caspase-3 Activity Assay

Caspase-3 activity was assessed using the CaspACE Assay System, Colorimetric (Promega, Madison, WI, USA) according to the manufacturer’s instructions. Briefly, PEL cells were pretreated with caspase inhibitor Ac-DEVD-CHO (5 μM) or antioxidant *N*-acetyl-cysteine (NAC, 10 mM) for 1 h or left untreated. PEL cells were further incubated with EGCG (20 μg/mL) for another 24 h, and lysed in cold lysis buffer provided by the manufacturer. Then 20 μg of total cell lysates were incubated with the caspase-3 substrate Ac-DEVD-p-nitroaniline (pNA) at 37 °C for 4 h. The chromophore pNA was released from the substrate upon cleavage by caspase-3, and free pNA was monitored by a spectrophotometer at 405 nm.

### 4.6. Acidic Vesicular Organelle (AVO) Staining

Acridine orange freely diffuses the membranes of cells and organelles. It is a marker of acidic vesicular organelles (AVOs) that fluoresces green in the whole cell except in acidic compartments (mainly late autophagosomes), where it fluoresces red. The development of AVOs is a typical feature of autophagy, and its formation indicates the maturation of autophagosomes and an efficient autophagic process, since only mature/late autophagosomes are acidic. The intensity of the red fluorescence is proportional to the number of AVOs in autophagic cells [37]. Following EGCG treatment, cell culture medium was removed from the cells and replaced with cell culture medium containing 5 ug/mL acridine orange, and then incubated for 10 min at 37 °C. Cells were then harvested, washed twice, and examined. The fluorescence was observed using a Nikon Eclipse TE2000-U inverted fluorescent microscope, with a 10×/0.30 NA Plan Fluor objective.

### 4.7. Intracellular Reactive Oxygen Species (ROS) Determination

The formation of ROS was measured by using a nonfluorescent probe, 2,7-diacetyl dichlorofluorescein (H_2_DCFDA), which can penetrate into the intracellular matrix of cells, where it is oxidized by ROS to form fluorescent dichlorofluorescein (DCF) [38]. PEL cells were untreated or treated with 20 μg/mL EGCG for 24 h. Following the drug treatment, cells were incubated with 5 μM H_2_DCFDA for 30 min at 37 °C and washed three times using PBS (1×), and then analyzed by flow cytometric analysis (FACS Calibur^TM^, BD Biosciences, Franklin Lakes, NJ, USA).

### 4.8. Measurement of Mitochondrial Membrane Potential

Mitochondrial membrane potential (MMP) levels were measured using Rhodamine 123 (Rh123) fluorescent dye (Sigma-Aldrich Chemical Co., Ex/Em = 485 nm/535 nm), a cationic fluorescent indicator that selectively accumulates within mitochondria in a membrane potential-dependent manner. Once the mitochondria membrane potential is lost, Rh123 is subsequently washed out of the cells. PEL cells were untreated or treated with 20 μg/mL EGCG for 24 h. Following the drug treatment, cells were incubated with 1 µg/mL Rh123 and incubated at 37 °C for 30 min in the dark. The samples were washed three times using PBS (1×), and then analyzed by flow cytometric analysis (FACS Calibur^TM^, BD Biosciences, Franklin Lakes, NJ, USA).

### 4.9. Western Blot Analysis

Total-protein extracts from the control and EGCG-treated BCBL-1 cells were analyzed by Western blot analysis, as described previously [39], using antibodies specific for Bax, p53, JNK, Ser^15^-phosphorylated p53, Thr^183^/Tyr^185^-phosphorylated JNK, Thr^183^/Tyr^185^-phosphorylated ERK1/2 and Thr^180^/Tyr^182^-phosphorylated p38 (Cell Signaling Technologies, Beverly, MA, USA), Beclin-1 and LC3B (GeneTex, Hsinchu, Taiwan), and β-actin (Sigma Chemical Co., St. Louis, MO, USA). In brief, BCBL-1 cells were untreated or treated with EGCG as described in the legends. The cells were then harvested by centrifugation and washed with cold PBS, and cell extracts were prepared in lysis buffer (0.5% Nonidet P-40, 50 mM Tris-HCl (pH 7.5), 0.25% sodium deoxycholate, 1 mM EDTA, 100 mM sodium chloride, 50 mM sodium fluoride, 500 μM sodium orthovanadate, 1× complete protease inhibitors (Roche)) for 60 min on ice. The lysate solution was spun at 12,000× *g* for 10 min at 4 °C, and supernatants were collected. Protein concentrations were assessed by Bradford assay before the samples were loaded. Equal amounts of proteins were separated by SDS–PAGE and transferred to a polyvinylidene difluoride (PVDF) membrane (Immobilon; Millipore, Billerica, MA, USA). Immunoblotting was performed with various antibodies and visualized using an enhanced chemiluminescence (Amersham, Arlington Heights, IL, USA) method.

### 4.10. Luciferase Reporter Assay

293T cells were co-transfected with the RTA promoter luciferase reporter plasmid [40] and the pTK-RL (encoding Renilla luciferase) control plasmid using Lipofectamine-2000 Transfection Reagent (Invitrogen). Cells were treated with EGCG at 20 h post-transfection and analyzed for luciferase activities at 24 h posttreatment. Luciferase activities were measured using the dual-luciferase reporter assay kit (Promega, Madison, WI, USA). Firefly luciferase activity from the RTA promoter reporter was normalized to the Renilla luciferase activity from the pTK-RL control plasmid. Relative fold activation compared to the control treatment. Relative luciferase activity was expressed as activation (%) relative to that of the reporter construct alone.

### 4.11. Reverse Transcription-PCR

Total RNA was extracted by using Trizol (Invitrogen) according to the manufacturer’s instructions. The purified mRNA samples were digested with DNase I (Promega) for 30 min at 37 °C, and the reaction was stopped by EDTA followed by heat inactivation at 70 °C. Reverse transcription-PCR (RT-PCR) was performed by using a SuperScript III (Invitrogen), and the synthesized cDNA samples were used as templates for PCRs using specific primers. Primers used were as followed: GAPDH forward: (CCCTTCATTGACCTCAACTA) and reverse: (CCAAAGTTGTCATGGATGAC); RTA forward: (TATCCAGGAAGCGGTCTCAT) and reverse: (GGGTTAAAGGGGATGATGCT); K7 forward: (AATATGGGAACACTGGAG) and reverse: (CTACAACTGGCCTGGAGA). The thermal cycle settings used on a Whatman Biometra T1 thermocycler (Göttingen, Germany) were as follows: 94 °C for 5 min as initial denaturation, 27 cycles of amplification (94 °C for 30 s, 56 °C for 30 s, and 72 °C for 45 s), followed by 72 °C for 10 min. PCR products were resolved on 1% agarose gel and the DNA was visualized by ethidium bromide.

### 4.12. Polymerase Chain Reaction (PCR) for Viral Load

BCBL-1 cells (2 × 10^5^/mL, 1 mL) were untreated or treated with EGCG or treated with 20 ng/mL phorbol-12-myristate-13-acetate (PMA, also known as TPA; Sigma) for 24 h. TPA was used to induce the production of viral particles. Then the cells stimulated by TPA were cultured in medium with or without EGCG (1 μg/mL and 5 μg/mL) for another 24 h. Then, 800 μL supernatant was centrifuged at 1200 rpm for 5 min followed by treatment with 20 U DNase I for 30 min at 37 °C to obtain only enveloped and encapsidated viral genomes. Viral DNA was extracted from 600 μL DNase-treated supernatant containing progeny viral particles using phenol/chloroform/isoamyl alcohol. Then, DNA was precipitated with 1/10 volume of 3 M sodium acetate, and one volume of absolute isopropanol followed by centrifugation at 13,000× *g* for 10 min at 4 °C. The extracted DNA samples were washed with 70% ethanol and dissolved in 10 μL sterile water. To quantify viral DNA, PCR was performed. The viral DNA was serially diluted. Each PCR mixture contained 2 μL viral DNA (1/100×, 1/10× and 1×). The viral DNA was amplified using the LANA primer set, forward: (AGCCCACCAGGAGATAATAC) and reverse: (TCATTTCCTGTGGAGAGTCC), and the RTA primer set. The PCR conditions were as follows: 94 °C for 5 min as initial denaturation, 32 cycles of amplification (94 °C for 30 s, 55 °C for 30 s, and 72 °C for 45 s), followed by 72 °C for 10 min. No amplification was observed in the no-template controls for the primer set.

### 4.13. Statistical Analysis

Unpaired *t*-test was adopted for statistical evaluation of the results. Significant differences were established at *p* < 0.05. All statistical analyses were performed in Prism Software (Graph Pad Prism 5, La Jolla, CA, USA).

## Supplementary Materials

Supplementary materials can be found at www.mdpi.com/1422-0067/19/1/16/s1.
