# Supplementary material for: Epigallocatechin-3-Gallate Suppresses Human Herpesvirus 8 Replication and Induces ROS Leading to Apoptosis and Autophagy in Primary Effusion Lymphoma Cells
Source: Int J Mol Sci. 2017 Dec 21;19(1):16. doi: 10.3390/ijms19010016 (PMC5795967; doi:10.3390/ijms19010016)
Supplement: Supplementary file 1 [file ijms-19-00016-s001.pdf]

International Journal of Molecular Sciences

Supplementary Materials

Epigallocatechin-3-Gallate Suppresses Human Herpesvirus 8 Replication and Induces ROS Leading to Apoptosis and Autophagy in Primary Effusion Lymphoma Cells

Ching-Yi Tsai [1], Chang-Yu Chen [1], Yee-Hsuan Chiou [2], Huey-Wen Shyu [1], Kuan-Hua Lin [1], Miao-Chen Chou [1], Mei-Han Huang [1] and Yi-Fen Wang [1,*]

[1] Department of Medical Laboratory Science and Biotechnology, Fooyin-University, Kaohsiung, Taiwan; a07646815@yahoo.com.tw (C.-Y.T.); chencyallen1689@gmail.com (C.-Y.C.); shyuhw@hotmail.com (H.-W.S.); su6473@mail2000.com.tw (K.-H.L.); miaochen119@gmail.com (M.-C.C.); hmhmhtw8@gmail.com (M.-H.H.)

[2] Department of Pediatrics, Kaohsiung Veterans General Hospital, Kaohsiung, Taiwan; chysn@ms6.hinet.net

* Correspondence: sc071@fy.edu.tw; Tel.: + ; Fax: +886-7-782-7162

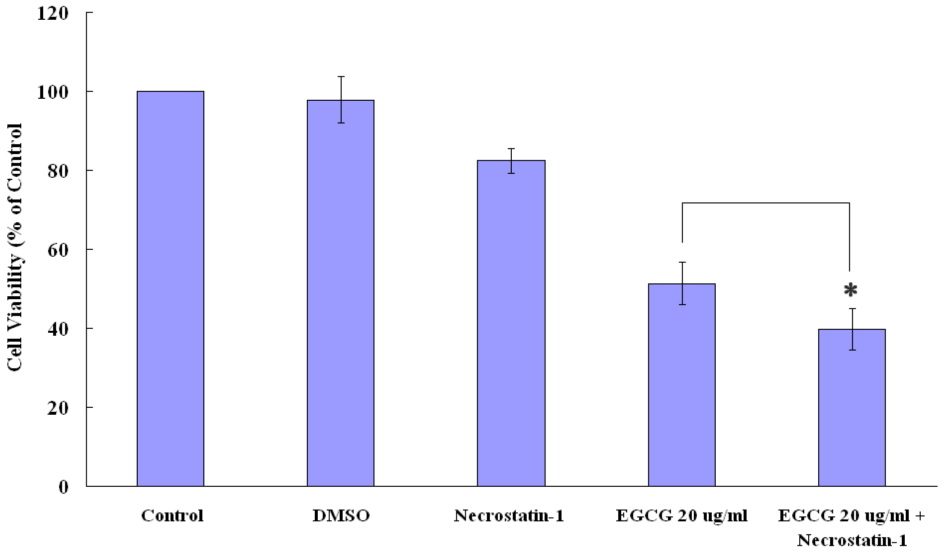

Figure S1. Effects of necroptosis inhibitor (necrostatin) on EGCG induced cell death of PEL cells. BCBL-1 cells were pretreated with necrostatin-1 1 h before EGCG (20 μg/ml) treatment. Cell viability was evaluated by trypan blue exclusion assay 24 h later. The cells in supplemented medium were used as control. The values represent mean ± SE of three independent experiments and are presented as the percentage of control. * $p < 0.05$ indicates significant differences between the cells treated with EGCG and the cells co-treated with EGCG and necrostatin-1.

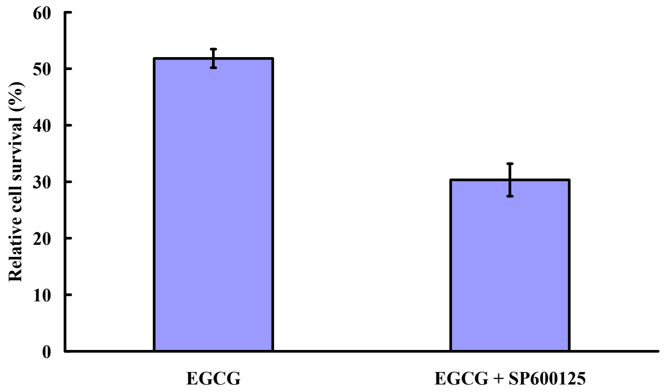

Figure S2. JNK inhibitor SP600125 enhanced the cytotoxicity of EGCG in HHV8 harboring PEL cells.
